# Temperature in the Friction Couple Consisting of Functionally Graded and Homogeneous Materials

**DOI:** 10.3390/ma15103600

**Published:** 2022-05-18

**Authors:** Aleksander Yevtushenko, Michał Kuciej, Katarzyna Topczewska, Przemysław Zamojski

**Affiliations:** Faculty of Mechanical Engineering, Bialystok University of Technology (BUT), 45C Wiejska Street, 15-351 Bialystok, Poland; a.yevtushenko@pb.edu.pl (A.Y.); m.kuciej@pb.edu.pl (M.K.); p.zamojski@pb.edu.pl (P.Z.)

**Keywords:** frictional heating, functionally graded materials, temperature, braking

## Abstract

An analytical model was developed to determine the temperature of friction coupling, in which one element was made of a functionally graded material (FGM) and the other was homogeneous. First, for such a system, the boundary–value problem of heat conduction was formulated with consideration of the heat generation due to friction. Then, using the Laplace integral transform, an exact solution to this problem was obtained for uniform sliding, and braking with constant deceleration. A numerical analysis was performed for the selected friction pair consisting of the FGM (zircon dioxide + titanium alloy) and cast iron. It was established that the use of elements made of a FGM consisting of ZrO_2_ and Ti-6Al-4V can significantly reduce the maximum temperature achieved in the friction system.

## 1. Introduction

Reviews of investigations on methods for establishing the temperature of systems containing friction elements made of functionally gradient materials (FGMs) can be found in previous articles [1,2,3]. In these studies, the methodology of determining the temperature in such friction couples under uniform sliding [1], during braking with time-dependent contact pressure [2], and considering the thermal sensitivity of component materials of FGMs was investigated [3]. The main factor in this methodology is an exact solution to the boundary–value heat conduction problem, taking into account the frictional heating of two semi-infinite bodies made of FGMs. It should be noted, however, that the obtained solutions did not allow determining automatically, with the help of limit transformations, solutions to the problems in the case when one of the friction pair elements is made of FGM and the other is homogeneous. Moreover, this type of friction pair is one of the most common [4]. Therefore, in this study, an attempt was made to develop a mathematical model for determining the temperature of a friction pair consisting of a body made of a two-component FGM, sliding on the surface of a homogeneous body. An exponential change in the thermal conductivity of the FGM with distance from the friction surface was assumed. Two modes of changing the sliding velocity over time were considered: uniform and linearly decreasing.

## 2. Statement of the Problem

**Figure 1:**
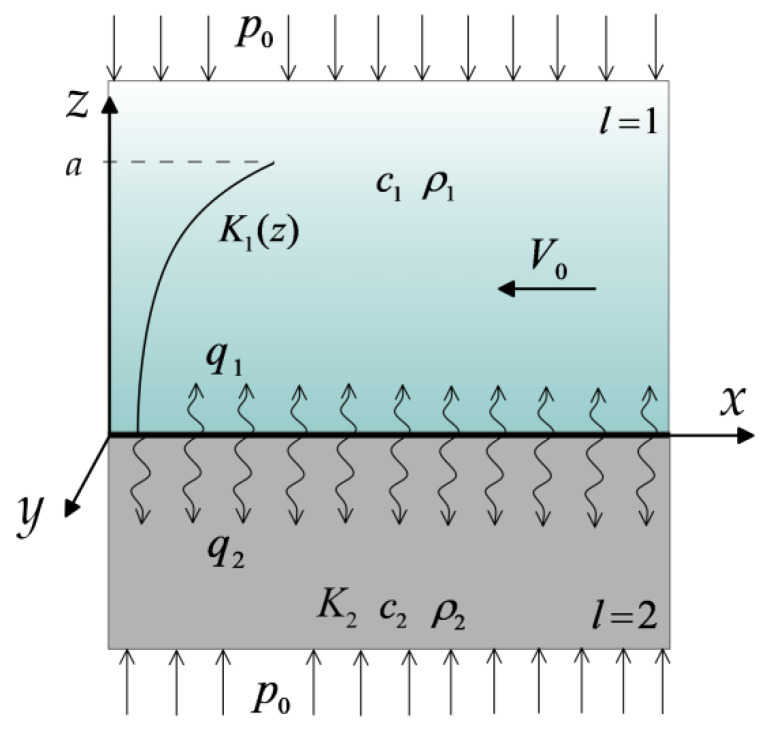
Scheme of the problem.

The object of study is the transient temperature field, initiated in the process of frictional heating of the friction pair elements of a braking system, corresponding to the brake pad and disc. Taking into account the fact that the heat generated as a result of friction during braking is mainly directed along the normal from the friction surface to the inside of both elements [5,6], for the description of the heating process of the system, a contact scheme of two semi-infinite bodies was adopted, related to the Cartesian coordinate system (Figure 1).

The pad (body 1) is made of a two-component functionally graded material (FGM), in such a way that the friction surface is a material of low thermal conductivity and high wear resistance (ceramics etc.), while the core material has high thermal conductivity (metal alloys, copper, iron etc.). The increase in thermal conductivity of the pad material in the distance from the friction surface is exponential. On the other hand, the disc (body 2) is made of a homogeneous material (cast iron etc.). A more detailed description of the adopted model assumptions is presented in our previous articles [1,2].

The analytical model presented in the manuscript concerns the frictional system of two semi-infinite bodies, in which it is not possible to take into consideration the heat exchange between the heated elements and the surrounding environment. It is known, however, that consideration of convection cooling, would lead to a lower maximum temperature; the most important parameters in the design process of frictional systems. It should be ensured that the theoretical value of the permissible temperature for a given material (i.e., the melting point) is not exceeded. For this reason, at the design stage, calculations should be performed for the maximum temperature achieved for adiabatic conditions on the free surfaces of the friction system.

The braking process with constant deceleration was considered when the contact pressure achieved its nominal value $p_{0}$ immediately at the beginning of the braking, with simultaneously reduction of velocity from the initial value $V_{0}$ to zero at the stopping moment $t=t_{s}$. For such braking, the specific friction power was written in the form:

(1) $$q(t)=q_{0}q^{*}(t),q_{0}=f_{0}p_{0}V_{0},q^{*}(t)=1-\frac{t}{t_{s}},\;0\leq t\leq t_{s},\;t_{s}=\frac{W_{0}}{q_{0}A_{a}},$$

where $f_{0}$—friction coefficient, $W_{0}$—initial kinetic energy of the system, and $A_{a}$— area of nominal contact between one brake pad and the disc.

The temperature field T(z,t) in the system consisting of two sliding semi-spaces was sought based on the solution to the following thermal problem of friction:

(2) $$\frac{\partial }{\partial z}\left[K_{1}(z)\frac{\partial T(z,t)}{\partial z}\right]=c_{1}\rho_{1}\frac{\partial T(z,t)}{\partial t},\;z>0,\;0<t\leq t_{s},$$

(3) $$K_{2}\frac{\partial^{2}T(z,t)}{\partial z^{2}}=c_{2}\rho_{2}\frac{\partial T(z,t)}{\partial t},\;z<0,\;0<t\leq t_{s},$$

(4) $$T(0^{+},t)=T(0^{-},t)\equiv T(t),\;0<t\leq t_{s},$$

(5) $${K_{2}\frac{\partial T(z,t)}{\partial z}|}_{z=0^{-}}-{K_{1}(z)\frac{\partial T(z,t)}{\partial z}|}_{z=0^{+}}=q(t),\;0<t\leq t_{s},$$

(6) $$T(z,t)\to T_{0},\;\left|z\right|\to \infty ,\;0<t\leq t_{s},$$

(7) $$T(z,0)=T_{0},\;\left|z\right|<\infty .$$

where

(8) $$K_{1}(z)=K_{1,1}e^{\gamma z},\;z\geq 0,\;\gamma \geq 0,$$

(9) $$c_{1}=c_{1,1}(1-v)+c_{1,2}v,\;\rho_{1}=\rho_{1,1}(1-v)+\rho_{1,2}v,\;0\leq v\leq 1,$$

temporal profile of specific friction power q(t) was determined from Equation (1), $K_{1,m}$, $c_{1,m}$, and $\rho_{1,m}$—thermal conductivity, specific heat, and density of the first (m=1) and the second (m=2) component of pad material, respectively, and parameters $K_{2}$, $c_{2}$, and $\rho_{2}$—correspond to the disc material, v—the relative volumetric fraction of the first component of the pad material, and $T_{0}$—temperature of the system at the initial time moment t=0.

The dimensionless variables and parameters were introduced:

(10) $$\zeta =\frac{z}{a},\;\tau =\frac{k_{1}t}{a^{2}},\;\tau_{s}=\frac{k_{1}t_{s}}{a^{2}},\;K^{*}=\frac{K_{2}}{K_{1,1}},\;k^{*}=\frac{k_{2}}{k_{1}},\;\Theta^{*}=\frac{T-T_{0}}{\Theta_{0}},\;\Theta_{0}=\frac{q_{0}a}{K_{1,1}^{\left(0\right)}},\;$$

where

(11) $$a=\sqrt{3k_{1}t_{s}},$$

(12) $$k_{1}=\frac{K_{1,1}}{c_{1}\rho_{1}},\;k_{2}=\frac{K_{2}}{c_{2}\rho_{2}}.$$

Taking into account the designations (10)–(12), the problem (2)–(9) was written in the form:

(13) $$\frac{\partial^{2}\Theta^{*}(\zeta ,\tau )}{\partial \zeta^{2}}+\gamma^{*}\frac{\partial \Theta^{*}(\zeta ,\tau )}{\partial \zeta }-e^{-\gamma^{*}\zeta }\frac{\partial \Theta^{*}(\zeta ,\tau )}{\partial \tau }=0,\;\zeta >0,\;0<\tau \leq \tau_{s},$$

(14) $$\frac{\partial^{2}\Theta^{*}(\zeta ,\tau )}{\partial \zeta^{2}}-\frac{1}{k^{*}}\frac{\partial \Theta^{*}(\zeta ,\tau )}{\partial \tau }=0,\;\zeta <0,\;0<\tau \leq \tau_{s},$$

(15) $$\Theta^{*}(0^{+},\tau )=\Theta^{*}(0^{-},\tau )\equiv \Theta^{*}(\tau ),\;0<\tau \leq \tau_{s},$$

(16) $${K^{*}\frac{\partial \Theta^{*}(\zeta ,\tau )}{\partial \zeta }|}_{\zeta =0^{-}}-{\frac{\partial \Theta^{*}(\zeta ,\tau )}{\partial \zeta }|}_{\zeta =0^{+}}=q^{*}(\tau ),\;0<\tau \leq \tau_{s},$$

(17) $$\Theta^{*}(\zeta ,\tau )\to 0,\;\left|\zeta \right|\to \infty ,\;0<\tau \leq \tau_{s},$$

(18) $$\Theta^{*}(\zeta ,0)=0,\;\left|\zeta \right|<\infty ,$$

where

(19) $$q^{*}(\tau )=1-\frac{\tau }{\tau_{s}},\;0<\tau \leq \tau_{s},$$

(20) $$\gamma^{*}\equiv a\gamma =\ln\left(\frac{K_{1,2}}{K_{1,1}}\right).$$

## 3. Frictional Heating under Uniform Sliding

First, the case of frictional heating process during sliding of the pad on the disc surface with constant velocity $V_{0}$ was considered. Then for $\tau_{s}\to \infty$ from the Equation (19), it follows that $q^{*}(\tau )=1$. For the boundary–value heat conduction problem (13)–(20) with a constant temporal profile of specific friction power $q^{*}(\tau )=1$, the integral Laplace transform was applied [7]:

(21) $${\bar{\Theta }}^{*}(\zeta ,p)\equiv L[\Theta^{*}(\zeta ,\tau );\;p]=\int_{0}^{\infty }\Theta^{*}(\zeta ,\tau )e^{-p\tau }d\tau ,$$

it was obtained:

(22) $$\frac{d^{2}{\bar{\Theta }}^{*}(\zeta ,p)}{d\zeta^{2}}+\gamma^{*}\frac{d{\bar{\Theta }}^{*}(\zeta ,p)}{d\zeta }-pe^{-\gamma^{*}\zeta }{\bar{\Theta }}^{*}(\zeta ,p)=0,\;\zeta >0,$$

(23) $$\frac{d^{2}{\bar{\Theta }}^{*}(\zeta ,p)}{d\zeta^{2}}-\frac{p}{k^{*}}{\bar{\Theta }}^{*}(\zeta ,p)=0,\;\zeta <0,$$

(24) $${\bar{\Theta }}^{*}(0^{+},p)={\bar{\Theta }}^{*}(0^{-},p)\equiv {\bar{\Theta }}^{*}(p),$$

(25) $${K^{*}\frac{d{\bar{\Theta }}^{*}(\zeta ,p)}{d\zeta }|}_{\zeta =0^{-}}-{\frac{d{\bar{\Theta }}^{*}(\zeta ,p)}{d\zeta }|}_{\zeta =0^{+}}=\frac{1}{p},$$

(26) $${\bar{\Theta }}^{*}(\zeta ,p)\to 0,\;\left|\zeta \right|\to \infty .$$

An exact solution to the ordinary differential Equations (22) and (23), which meet the boundary conditions (24)–(26) has the form:

(27) $${\bar{\Theta }}^{*}(\zeta ,p)=\frac{\Delta_{1}(\zeta ,p)}{p\sqrt{p\;}\Delta (p)},\;\zeta \geq 0,\;{\bar{\Theta }}^{*}(\zeta ,p)=\frac{\Delta_{2}(\zeta ,p)}{p\sqrt{p\;}\Delta (p)},\;\zeta \leq 0,$$

where

(28) $$\Delta_{1}(\zeta ,p)=e^{-0.5\gamma^{*}\zeta }I_{1}\left(\frac{2}{\gamma^{*}}\sqrt{p}e^{-0.5\gamma^{*}\zeta }\right),\;\Delta_{2}(\zeta ,p)=e^{\sqrt{\frac{p}{k^{*}}}\;\zeta }I_{1}\left(\frac{2}{\gamma^{*}}\sqrt{p}\right),$$

(29) $$\Delta (p)=I_{0}\left(\frac{2}{\gamma^{*}}\sqrt{p}\right)+K_{\varepsilon }I_{1}\left(\frac{2}{\gamma^{*}}\sqrt{p}\right),$$

$I_{k}(x)$—modified Bessel functions of the first kind of the *k*th order k=0, 1 [8].

Using the inverse Laplace transform to the solution (27)–(29), the dimensional temperature rise was found in the form:

(30) Θ∗(ζ,τ)≡L−1[Θ¯∗(ζ,p); τ]=12πi∫ω−i∞ω+i∞Θ¯∗(ζ,p)epτdp, τ≥0, ω≡Rep>0, i≡−1.

**Figure 2:**
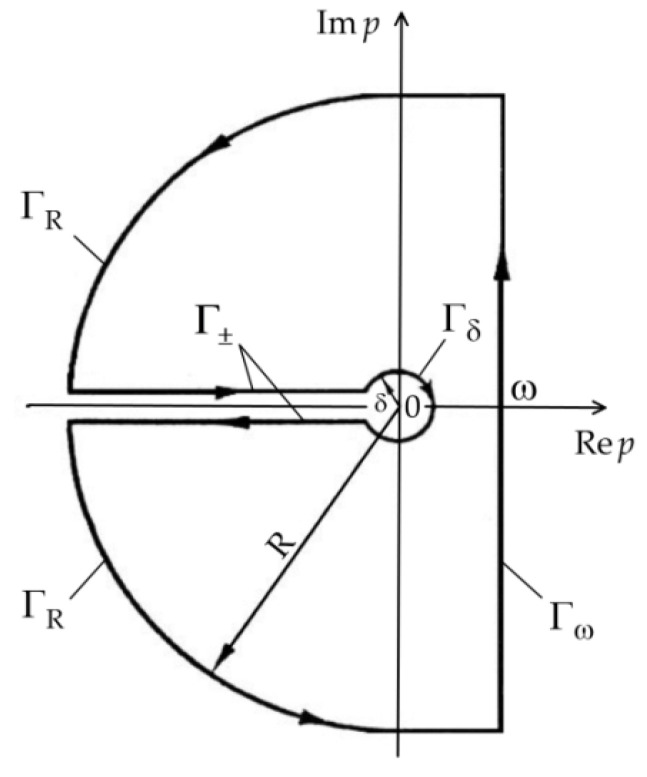
Integration contour Γ.

The presence of $\sqrt{p}$, as well as the lack of the roots of function Δ(p), testifies that the solution (36)–(39) has a branch point for p=0. Therefore, to perform the integration on the complex plane (Rep, Im p), the closed contour Γ was chosen, as demonstrated in Figure 2. The contour Γ consists of the straight line $\Gamma_{\omega }$ Re p=ω, the circles $\Gamma_{R}$ and $\Gamma_{\delta }$ with the radii R and δ, respectively, with the center p=0, and a cut of a complex *p*–plane along negative real axis Re p<0 and two boundaries $\Gamma_{\pm }$. Within the contour Γ, the integral function ${\bar{\Theta }}^{*}(\zeta ,p)$ in the Equation (30) is unambiguous and analytical.

Then, based on Cauchy’s theorem we obtained [9]:

(31) $$\frac{1}{2\pi i}\oint_{\Gamma }{\bar{\Theta }}^{*}(\zeta ,p)e^{p\tau }dp=0.$$

Since the transform ${\bar{\Theta }}^{*}(\zeta ,\tau )$ carries out the conditions of Jordan’s lemma [7]:

(32) $$\left|\frac{\Delta_{l}(\zeta ,p)}{p\sqrt{p}\Delta (p)}\right|\leq \frac{const.}{p\sqrt{p}},\;l=1,2,$$

integrands on arcs $\Gamma_{R}$ in the Equation (31) tend to zero for R→∞; therefore, on the basis of the relations (30) and (31), the dimensional temperature rise was written in the form:

(33) $$\Theta^{*}(\zeta ,\tau )+\Theta_{+}^{*}(\zeta ,\tau )+\Theta_{-}^{*}(\zeta ,\tau )+\Theta_{\delta }^{*}(\zeta ,\tau )=0,\;\left|\zeta \right|<\infty ,\;\tau \geq 0,$$

where

(34) $$\Theta_{\pm }^{*}(\zeta ,\tau )=\frac{1}{2\pi i}\int_{\Gamma_{\pm }}{\bar{\Theta }}^{*}(\zeta ,p)e^{p\tau }dp,\;\Theta_{\delta }^{*}(\zeta ,\tau )=\frac{1}{2\pi i}\int_{\Gamma_{\delta }}{\bar{\Theta }}^{*}(\zeta ,p)e^{p\tau }dp.$$

In the polar coordinate system (r,φ) with center in the point p=0, parameter of the Laplace transform $p=re^{i\varphi }$, r≥0, and |φ|≤π. Then on the boundary $\Gamma_{+}$ we obtained $p=re^{i\pi }=-r$, $\sqrt{p}=i\sqrt{r}$, and on the edge $\Gamma_{-}$, respectively, $p=re^{-i\pi }=-r$, $\sqrt{p}=-i\sqrt{r}$ and the first two integrals (34) took the form:

(35) $$\Theta_{\pm }^{*}(\zeta ,\tau )=\pm \frac{1}{2\pi i}\int_{0}^{\infty }{\bar{\Theta }}_{\pm }^{*}(\zeta ,r)e^{-r\tau }dr,\;\left|\zeta \right|<\infty ,\;\tau \geq 0,$$

where ${\bar{\Theta }}_{\pm }^{*}(\zeta ,r)\equiv {\bar{\Theta }}^{*}(\zeta ,re^{\pm i\pi })$.

Taking into account the dependencies [8]:

(36) $$I_{0}(x)=J_{0}(ix),\;I_{1}(x)=-iJ_{1}(ix),$$

(where $J_{k}(x)$ are the Bessel functions of the first kind of the *k*th order k=0,1), from Equations (27)–(29) was obtained:

(37) $${\bar{\Theta }}_{\pm }^{*}(\zeta ,r)=\frac{\Delta_{1}^{\pm }(\zeta ,r)}{r\sqrt{r}\;\Delta^{\mp }(r)},\;\zeta \geq 0,\;{\bar{\Theta }}_{\pm }^{*}(\zeta ,r)=\frac{\Delta_{2}^{\pm }(\zeta ,r)}{r\sqrt{r}\;\Delta^{\mp }(r)},\;\zeta \leq 0,$$

where:

(38) $$\Delta_{1}^{\pm }(\zeta ,r)=\pm ie^{-0.5\gamma^{*}\zeta }J_{1}\left(\frac{2}{\gamma^{*}}\sqrt{r}e^{-0.5\gamma^{*}\zeta }\right),\;\Delta_{2}^{\pm }(\zeta ,r)=\pm ie^{\pm \;i\sqrt{\frac{r}{k^{*}}}\zeta }J_{1}\left(\frac{2}{\gamma^{*}}\sqrt{r}\right),$$

(39) $$\Delta^{\pm }(\zeta ,r)=K_{\varepsilon }J_{1}\left(\frac{2}{\gamma^{*}}\sqrt{r}\right)\pm iJ_{0}\left(\frac{2}{\gamma^{*}}\sqrt{r}\right).$$

On the circle $\Gamma_{\delta }$ it is $p=\delta e^{i\varphi }$, $\sqrt{p}=\sqrt{\delta }e^{0.5i\varphi }$, |φ|≤π. Approaching the limit δ→0 with consideration of the solutions forms (27)–(29), the third integral (34) was written as:

(40) $$\Theta_{\delta }^{*}(\zeta ,\tau )=\underset{\delta \to 0}{\lim}\left(-\frac{1}{2\pi i}\int_{-\pi }^{\pi }{\bar{\Theta }}_{\delta }^{*}(\zeta ,\delta e^{i\varphi })e^{\delta e^{i\varphi }\tau }i\delta e^{i\varphi }d\varphi \right),\;\tau \geq 0,$$

where

(41) $${\bar{\Theta }}_{\delta }^{*}(\zeta ,\delta e^{i\varphi })=\frac{\Delta_{1}(\zeta ,\delta e^{i\varphi })}{\delta \sqrt{\delta }e^{1.5i\varphi }\Delta (\delta e^{i\varphi })},\zeta \geq 0,\;{\bar{\Theta }}_{\delta }^{*}(\zeta ,\delta e^{i\varphi })=\frac{\Delta_{2}(\zeta ,\delta e^{i\varphi })}{\delta \sqrt{\delta }e^{1.5i\varphi }\Delta (\delta e^{i\varphi })},\zeta \leq 0,$$

(42) $$\Delta_{1}(\zeta ,\delta e^{i\varphi })=e^{-0.5\gamma^{*}\zeta }I_{1}\left(\frac{2}{\gamma^{*}}\sqrt{\delta }e^{0.5i\varphi }e^{-0.5\gamma^{*}\zeta }\right),$$

(43) $$\Delta_{2}(\zeta ,\delta e^{i\varphi })=e^{\sqrt{\frac{\delta }{k^{*}}}\;\zeta \;e^{0.5i\varphi }}I_{1}\left(\frac{2}{\gamma^{*}}\sqrt{\delta }e^{0.5i\varphi }\right),$$

(44) $$\Delta^{\pm }(\zeta ,r)=K_{\varepsilon }J_{1}\left(\frac{2}{\gamma^{*}}\sqrt{r}\right)\pm iJ_{0}\left(\frac{2}{\gamma^{*}}\sqrt{r}\right).$$

Substituting the functions (41)–(44) into Equation (40), it was found:

(45) $$\Theta_{\delta }^{*}(\zeta ,\tau )=\underset{\delta \to 0}{\lim}\left(-\frac{1}{2\pi }\int_{-\pi }^{\pi }\frac{\Delta_{1}(\zeta ,\delta e^{i\varphi })}{\sqrt{\delta }e^{0.5i\varphi }\Delta (\delta e^{i\varphi })}e^{\delta e^{i\varphi }\tau }d\varphi \right),\;\zeta \geq 0,\;\tau \geq 0,$$

(46) $$\Theta_{\delta }^{*}(\zeta ,\tau )=\underset{\delta \to 0}{\lim}\left(-\frac{1}{2\pi }\int_{-\pi }^{\pi }\frac{\Delta_{2}(\zeta ,\delta e^{i\varphi })}{\sqrt{\delta }e^{0.5i\varphi }\Delta (\delta e^{i\varphi })}e^{\delta e^{i\varphi }\tau }d\varphi \right),\;\zeta \leq 0,\;\tau \geq 0.$$

For small values of the argument [8]:

(47) $$I_{0}(x)≅1,\;I_{1}(x)≅0.5x,$$

from Equations (45) and (46), the following was obtained:

(48) $$\Theta_{\delta }^{*}(\zeta ,\tau )=-\frac{1}{\gamma^{*}}e^{-0.5\gamma^{*}\zeta },\zeta \geq 0,\;\Theta_{\delta }^{*}(\zeta ,\tau )=-\frac{1}{\gamma^{*}},\zeta \leq 0,\;\tau \geq 0.$$

Applying the function $\Theta_{\pm }^{*}(\zeta ,\tau )$ (35), (37)–(39), and $\Theta_{\delta }^{*}(\zeta ,\tau )$ (48) into the Equation (33) and introducing the notation: $\sqrt{r}=x$, $r=x^{2}$, the dimensional rise of temperature was found in the form:

(49) $$\Theta^{*}(\zeta ,\tau )=\frac{1}{\gamma^{*}}\left[e^{-0.5\gamma^{*}\zeta }-\frac{4}{\pi }\int_{0}^{\infty }F(x)G_{1}(\zeta ,x)e^{-{\left(0.5\gamma^{*}x\right)}^{2}\tau }dx\right],\;\zeta \geq 0,\;\tau \geq 0,$$

(50) $$\Theta^{*}(\zeta ,\tau )=\frac{1}{\gamma^{*}}\left[1-\frac{4}{\pi }\int_{0}^{\infty }F(x)G_{2}(\zeta ,x)e^{-{\left(0.5\gamma^{*}x\right)}^{2}\tau }dx\right],\;\zeta \leq 0,\;\tau \geq 0,$$

where

(51) $$F(x)=\frac{J_{1}(x)}{x^{2}\{{\left[J_{0}(x)\right]}^{2}+{\left[K_{\varepsilon }J_{1}(x)\right]}^{2}\}},$$

(52) $$G_{1}(\zeta ,x)=K_{\varepsilon }e^{-0.5\gamma^{*}\zeta }J_{1}(xe^{-0.5\gamma^{*}\zeta }),$$

(53) $$G_{2}(\zeta ,x)=K_{\varepsilon }J_{1}(x)\cos\left(\frac{\gamma^{*}\zeta }{2\sqrt{k^{*}}}x\right)-J_{0}(x)\sin\left(\frac{\gamma^{*}\zeta }{2\sqrt{k^{*}}}x\right).$$

Substituting ζ=0 into Equations (49)–(53) it was established that the temperature rise on the contact surface included in the boundary condition (24) has the form:

(54) $$\Theta^{*}(\tau )=\frac{1}{\gamma^{*}}\left[1-\frac{4}{\pi }\int_{0}^{\infty }G(x)e^{-{\left(0.5\gamma^{*}x\right)}^{2}\tau }dx\right],\;\tau \geq 0,$$

where

(55) $$G(x)=\frac{K_{\varepsilon }{\left[J_{1}(x)\right]}^{2}}{x^{2}\{{\left[J_{0}(x)\right]}^{2}+{\left[K_{\varepsilon }J_{1}(x)\right]}^{2}\}}.$$

On the basis of the Fourier’s law, the intensities of heat fluxes directed from the contact surface towards the inside of the friction pair elements were defined:

(56) $$q_{1}(t)={-K_{1,1}\frac{\partial \Theta (z,t)}{\partial z}|}_{z=0^{+}},\;q_{2}(t)={K_{2}\frac{\partial \Theta (z,t)}{\partial z}|}_{z=0^{-}},\;t\geq 0,$$

The dimensionless form of dependencies (56) can be found as:

(57) $$q_{l}^{*}(\tau )=\frac{q_{l}(t)}{q_{0}},\;l=1,2,$$

and taking account of (8) and (18), it was obtained:

(58) $$q_{1}^{*}(\tau )={-\frac{\partial \Theta^{*}(\zeta ,\tau )}{\partial \zeta }|}_{\zeta =0^{+}},\;q_{2}^{*}(\tau )={K^{*}\frac{\partial \Theta^{*}(\zeta ,\tau )}{\partial \zeta }|}_{\zeta =0^{-}},\;\tau \geq 0.$$

Substituting the dimensionless temperature rise (49)–(53) into Equation (58) and differentiating, it was found:

(59) $$q_{1}^{*}(\tau )=1+\frac{2}{\pi }\int_{0}^{\infty }Q(x)e^{-{\left(0.5\gamma^{*}x\right)}^{2}\tau }dx,\;q_{2}^{*}(\tau )=-\frac{2}{\pi }\int_{0}^{\infty }Q(x)e^{-{\left(0.5\gamma^{*}x\right)}^{2}\tau }dx,\;\tau \geq 0,$$

where

(60) $$Q(x)=\frac{K_{\varepsilon }J_{0}(x)J_{1}(x)}{x\{{\left[J_{0}(x)\right]}^{2}+{\left[K_{\varepsilon }J_{1}(x)\right]}^{2}\}}.$$

From Equations (59) and (60) it follows that $q_{1}^{*}(\tau )+q_{2}^{*}(\tau )=1$, which confirms the fulfillment of the boundary condition (16) for $q^{*}(\tau )=1,\;\tau \geq 0$.

## 4. Asymptotic Solutions

It should be noted that solutions (49)–(55) have the form of a quadrature; thus, using them, numerical integration should be performed each time on the range of bounded fields. However, in the case of small and large values of dimensionless time τ (Fourier number), the corresponding asymptotic solution will be obtained in the analytical form, not requiring numerical integration.

*Small values of the Fourier number*0≤τ<<1 (large values of the parameter p of the Laplace integral transform (30)). At large values of arguments, the modified Bessel functions behave as follows [8]:

(61) $$I_{0}(x)≅\frac{e^{x}}{\sqrt{2\pi x}}\left(1+\frac{1}{8x}+\frac{9}{128x^{2}}+\ldots \right),\;I_{1}(x)≅\frac{e^{x}}{\sqrt{2\pi x}}\left(1-\frac{3}{8x}-\frac{15}{128x^{2}}-\ldots \right).$$

Limiting only to the first two components in the formula (61), the transforms of the dimensionless temperature rise (27)–(29) were written in the form:

(62) $${\bar{\Theta }}^{*}(\zeta ,p)≅\frac{e^{-0.25\gamma^{*}\zeta -\alpha \sqrt{p}}}{(1+K_{\varepsilon })p\sqrt{p}}\left(1-\frac{3\gamma^{*}e^{0.5\gamma^{*}\zeta }}{16\sqrt{p}}\right){\left[1+\frac{\gamma^{*}(1-3K_{\varepsilon })}{16(1+K_{\varepsilon })\sqrt{p}}\right]}^{-1},\;\zeta \geq 0,$$

(63) $${\bar{\Theta }}^{*}(\zeta ,p)≅\frac{e^{\sqrt{\frac{p}{k^{*}}\;}\zeta }}{(1+K_{\varepsilon })p\sqrt{p}}\left(1-\frac{3\gamma^{*}}{16\sqrt{p}}\right){\left[1+\frac{\gamma^{*}(1-3K_{\varepsilon }}{16(1+K_{\varepsilon })\sqrt{p}}\right]}^{-1},\;\zeta \leq 0,$$

where

(64) $$\alpha =\frac{2}{\gamma^{*}}(1-e^{-0.5\gamma^{*}\zeta }),\;\zeta \geq 0.$$

Taking into consideration that:

(65) $$\left(1-\frac{3\gamma^{*}e^{0.5\gamma^{*}\zeta }}{16\sqrt{p}}\right){\left[1+\frac{\gamma^{*}(1-3K_{\varepsilon }}{16(1+K_{\varepsilon })\sqrt{p}}\right]}^{-1}\approx 1-\frac{\gamma^{*}}{16\sqrt{p}}\left(3e^{0.5\gamma^{*}\zeta }+\frac{1-3K_{\varepsilon }}{1+K_{\varepsilon }}\right),$$

(66) $$\left(1-\frac{3\gamma^{*}}{16\sqrt{p}}\right){\left[1+\frac{\gamma^{*}(1-3K_{\varepsilon }}{16(1+K_{\varepsilon })\sqrt{p}}\right]}^{-1}\approx 1-\frac{\gamma^{*}}{4(1+K_{\varepsilon })\sqrt{p}},$$

the transforms (62)–(64) were obtained in the form:

(67) $${\bar{\Theta }}^{*}(\zeta ,p)≅\frac{e^{-0.25\gamma^{*}\zeta -\alpha \sqrt{p}}}{(1+K_{\varepsilon })p\sqrt{p}}\left[1-\frac{\gamma^{*}}{16\sqrt{p}}\left(3e^{0.5\gamma^{*}\zeta }+\frac{1-3K_{\varepsilon }}{1+K_{\varepsilon }}\right)\right],\;\zeta \geq 0,$$

(68) $${\bar{\Theta }}^{*}(\zeta ,p)≅\frac{e^{\sqrt{\frac{p}{k^{*}}\;}\zeta }}{(1+K_{\varepsilon })p\sqrt{p}}\left(1-\frac{\gamma^{*}}{4(1+K_{\varepsilon })\sqrt{p}}\right),\;\zeta \leq 0.$$

Taking account of the relations [10]:

(69) $$L^{-1}\left[\frac{e^{-a\sqrt{p}}}{p\sqrt{p^{n}}};\tau \right]={\left(4\tau \right)}^{\frac{n}{2}}i^{n}\mathrm{erfc}\left(\frac{a}{2\sqrt{\tau }}\right),\;n=1,2,\;a\geq 0,$$

from the transforms of solutions (67) and (68), the dimensionless temperature rises were found:

(70) $$\begin{array}{c} \Theta^{*}(\zeta ,\tau )≅\frac{2e^{-0.25\gamma^{*}\zeta }\sqrt{\tau }}{\left(1+K_{\varepsilon }\right)}\left[\mathrm{ierfc}\left(\frac{\alpha }{2\sqrt{\tau }}\right)-\frac{\gamma^{*}\sqrt{\tau }}{8}\left(3e^{0.5\gamma^{*}\zeta }+\frac{1-3K_{\varepsilon }}{1+K_{\varepsilon }}\right)i^{2}\mathrm{erfc}\left(\frac{\alpha }{2\sqrt{\tau }}\right)\right],\\ \zeta \geq 0, \end{array}$$

(71) $$\begin{array}{c} \Theta^{*}(\zeta ,\tau )≅\frac{2\sqrt{\tau }}{\left(1+K_{\varepsilon }\right)}\left[\mathrm{ierfc}\left(\frac{\left|\zeta \right|}{2\sqrt{k^{*}\tau }}\right)-\frac{\gamma^{*}\sqrt{\tau }}{2(1+K_{\varepsilon })}i^{2}\mathrm{erfc}\left(\frac{\left|\zeta \right|}{2\sqrt{k^{*}\tau }}\right)\right],\;\zeta \leq 0,\\ 0\leq \tau <<1, \end{array}$$

where

(72) $$\begin{array}{c} i^{2}\mathrm{erfc}(x)=0.25[\mathrm{erfc}(x)-2x\;\mathrm{ierfc}(x)],\;\mathrm{ierfc}(x)=\pi^{-0.5}e^{-x^{2}}-x\;\mathrm{erfc}(x),\\ \mathrm{erfc}(x)=1-\mathrm{erf}(x), \end{array}$$

erf(x)—Gauss error function [8]. On the contact surface ζ=0 from Equations, (70) and (71) it was obtained:

(73) $$\Theta^{*}(\tau )≅\frac{2\sqrt{\tau }}{\left(1+K_{\varepsilon }\right)}\left[\frac{1}{\sqrt{\pi }}-\frac{\gamma^{*}\sqrt{\tau }}{8(1+K_{\varepsilon })}\right],\;0\leq \tau <<1.$$

Approaching in Equations (70)–(73) the limit $\gamma^{*}\to 0$ (α→ζ), the solution for homogeneous materials was obtained [11]:

(74) $$\Theta^{*}(\zeta ,\tau )≅\frac{2\sqrt{\tau }}{\left(1+K_{\varepsilon }\right)}\mathrm{ierfc}\left(\frac{\zeta }{2\sqrt{\tau }}\right),\;\zeta \geq 0,\;$$

(75) $$\Theta^{*}(\zeta ,\tau )≅\frac{2\sqrt{\tau }}{\left(1+K_{\varepsilon }\right)}\mathrm{ierfc}\left(\frac{\left|\zeta \right|}{2\sqrt{k^{*}\tau }}\right),\;\zeta \leq 0,$$

(76) $$\Theta^{*}(\tau )≅\frac{2}{\left(1+K_{\varepsilon }\right)}\sqrt{\frac{\tau }{\pi }\;},\;0\leq \tau <<1.$$

*Large values of Fourier number*τ>>1(small values of the parameter p of the Laplace integral transform (30)). Distributions of the modified Bessel functions for small values of argument in the power series have the form [8]:

(77) $$I_{0}(x)≅1+\frac{x^{2}}{4}+\ldots ,\;I_{1}(x)≅\frac{x}{2}\left(1+\frac{x^{2}}{8}+\ldots \right).$$

Taking into account the relations (77), the Laplace transforms of dimensionless temperature rise (27)–(29) were written as:

(78) $${\bar{\Theta }}^{*}(\zeta ,p)≅\frac{e^{-\gamma^{*}\zeta }}{\gamma^{*}}\left[\frac{\beta }{p(\beta +\sqrt{p})}+\frac{e^{-\gamma^{*}\zeta }}{2K_{\varepsilon }\gamma^{*}(\beta +\sqrt{p}}\right],\;\zeta \geq 0,$$

(79) $${\bar{\Theta }}^{*}(\zeta ,p)≅\frac{e^{\sqrt{\frac{p}{k^{*}}\;}\zeta }}{\gamma^{*}}\left[\frac{\beta }{p(\beta +\sqrt{p})}+\frac{1}{2K_{\varepsilon }\gamma^{*}(\beta +\sqrt{p}}\right],\;\zeta \leq 0,$$

where

(80) $$\beta =\frac{\gamma^{*}}{K_{\varepsilon }}.$$

Using the dependencies [10]:

(81) $$L^{-1}\left[\frac{e^{-a\sqrt{p}}}{\left(\beta +\sqrt{p}\right)};\tau \right]=\frac{e^{-\frac{a^{2}}{4\tau }}}{\sqrt{\pi \tau }}-\beta \;e^{a\beta +\beta^{2}\tau }\mathrm{erfc}\left(\frac{a}{2\sqrt{\tau }}+\beta \sqrt{\tau }\right),$$

(82) $$L^{-1}\left[\frac{\beta \;e^{-a\sqrt{p}}}{p(\beta +\sqrt{p})};\tau \right]=\mathrm{erfc}\left(\frac{a}{2\sqrt{\tau }}\right)-e^{a\beta +\beta^{2}\tau }\mathrm{erfc}\left(\frac{a}{2\sqrt{\tau }}+\beta \sqrt{\tau }\right),\;a\geq 0,$$

from the transform solutions (78) and (79), the dimensionless temperature rises were obtained in the form:

(83) $$\Theta^{*}(\zeta ,\tau )≅\frac{e^{-\gamma^{*}\zeta }}{\gamma^{*}}\left\{1-e^{\beta^{2}\tau }\mathrm{erfc}(\beta \sqrt{\tau })+\frac{e^{-\gamma^{*}\zeta }}{2K_{\varepsilon }\gamma^{*}}\left[\frac{1}{\sqrt{\pi \tau }}-\beta \;e^{\beta^{2}\tau }\mathrm{erfc}(\beta \sqrt{\tau })\right]\right\},\;\zeta \geq 0,\;\tau >>1,$$

(84) $$\begin{array}{l} \Theta^{*}(\zeta ,\tau )≅\frac{1}{\gamma^{*}}\{\mathrm{erfc}\left(\frac{\left|\zeta \right|}{2\sqrt{k^{*}\tau }}\right)-e^{\frac{\beta \left|\zeta \right|}{\sqrt{k^{*}}}+\beta^{2}\tau }\mathrm{erfc}\left(\frac{\left|\zeta \right|}{2\sqrt{k^{*}\tau }}+\beta \sqrt{\tau }\right)+\\ \;\;\;\;\;\;\;\;\;\;\;+\frac{1}{2K_{\varepsilon }\gamma^{*}}\left[\frac{e^{-\frac{\zeta^{2}}{4k^{*}\tau }}}{\sqrt{\pi \tau }}-\beta e^{\frac{\beta \left|\zeta \right|}{\sqrt{k^{*}}}+\beta^{2}\tau }\mathrm{erfc}\left(\frac{\left|\zeta \right|}{2\sqrt{k^{*}\tau }}+\beta \sqrt{\tau }\right)\right]\},\;\;\zeta \leq 0,\;\;\tau >>1. \end{array}$$

Substituting ζ=0 into Equations (83) and (84), it was found:

(85) $$\Theta^{*}(\tau )≅\frac{1}{\gamma^{*}}\left\{1-e^{\beta^{2}\tau }\mathrm{erfc}(\beta \sqrt{\tau })+\frac{1}{2K_{\varepsilon }\gamma^{*}}\left[\frac{1}{\sqrt{\pi \tau }}-\beta \;e^{\beta^{2}\tau }\mathrm{erfc}(\beta \sqrt{\tau })\right]\right\},\;\tau >>1.$$

## 5. Temperature Field during Braking with Constant Deceleration

Based on Duhamel’s theorem [12], the dimensionless temperature rise during braking with constant deceleration was sought in the form:

(86) $${\hat{\Theta }}^{*}(\zeta ,\tau )=\frac{\partial }{\partial \tau }\int_{0}^{\tau }q^{*}(\tau -s)\Theta^{*}(\zeta ,s)ds,\;\left|\zeta \right|<\infty ,\;0\leq \tau \leq \tau_{s},$$

where the temporal profiles of the specific friction power $q^{*}(\tau )$ and function $\Theta^{*}(\zeta ,\tau )$ were determined from Equations (19), (49), and (50), respectively. Performing the integration first, and then differentiating, from the Equation (86) we obtained:

(87) $${\hat{\Theta }}^{*}(\zeta ,\tau )=\frac{1}{\gamma^{*}}\left[e^{-0.5\gamma^{*}\zeta }q^{*}(\tau )-\frac{4}{\pi }\int_{0}^{\infty }F(x)G_{1}(\zeta ,x)P(\tau ,x)dx\right],\;\zeta \geq 0,\;0\leq \tau \leq \tau_{s},$$

(88) $${\hat{\Theta }}^{*}(\zeta ,\tau )=\frac{1}{\gamma^{*}}\left[1-\frac{4}{\pi }\int_{0}^{\infty }F(x)G_{2}(\zeta ,x)P(\tau ,x)dx\right],\;\zeta \leq 0,\;0\leq \tau \leq \tau_{s},$$

where

(89) $$P(\tau ,x)=e^{-{\left(0.5\gamma^{*}x\right)}^{2}\tau }-\frac{\left(1-e^{-{\left(0.5\gamma^{*}x\right)}^{2}\tau }\right)}{{\left(0.5\gamma^{*}x\right)}^{2}\tau_{s}},$$

and functions F(x), $G_{1}(\zeta ,x)$, and $G_{2}(\zeta ,x)$ can be found from the formulas (51)–(53).

The temperature change on the friction surface was found, substituting ζ=0 into the Equations (87) and (88), in the form:

(90) $${\hat{\Theta }}^{*}(\tau )=\frac{1}{\gamma^{*}}\left[1-\frac{4}{\pi }\int_{0}^{\infty }G(x)P(\tau ,x)dx\right],\;\tau \geq 0,$$

where functions G(x) and P(τ,x) were determined from relations (55) and (89), respectively.

Knowing the dimensionless temperature rise (87), (88), from formulas (58) the dimensionless intensities of frictional heat fluxes were found:

(91) $${\hat{q}}_{1}^{*}(\tau )=q^{*}(\tau )+\frac{2}{\pi }\int_{0}^{\infty }Q(x)P(\tau ,x)dx,\;{\hat{q}}_{2}^{*}(\tau )=-\frac{2}{\pi }\int_{0}^{\infty }Q(x)P(\tau ,x)dx,\;0\leq \tau \leq \tau_{s},$$

where functions Q(x) and P(τ,x) have the forms (60) and (89), respectively. From Equation (91) it follows that ${\hat{q}}_{1}^{*}(\tau )+{\hat{q}}_{2}^{*}(\tau )=q^{*}(\tau )$, which confirms the fulfillment of the boundary condition (16) with the dimensionless specific friction power $q^{*}(\tau )$ in the form (19).

## 6. Numerical Analysis

Calculations were performed for a friction pair, where the first element (pad) is made of two-component FGM: zircon dioxide ZrO_2_ (friction surface) and titanium alloy Ti−6Al−4V(core). While the second material (brake disc) is homogeneous: cast iron ChNMKh. The properties of the materials are included in Table 1.

The values of the remaining input parameters used to perform the calculations are listed in Table 2.

Then, from formulas (1) and (19), the nominal value of specific friction power $q_{0}=3.87\;\mathrm{MW}\;m^{-2}$, braking time $t_{s}=12.1\;s$, and gradient parameter $\gamma^{*}=1.26$ were determined. Based on Equation (9), for an equal volumetric fraction of FGM component (v=0.5), the effective values of specific heat capacity and density of the pad material were obtained, $c_{1}=495.45\;J\;{\mathrm{kg}}^{-1}K^{-1}$, $\rho_{1}=5266.97\;\mathrm{kg}\;m^{-3}$, respectively. Thereafter, the following parameters were calculated sequentially: thermal diffusivity $k_{1}=0.743\cdot 10^{-6}\;m^{2}s^{-1}$ and $k_{2}=1.65\cdot 10^{-5}\;m^{2}s^{-1}$, the effective depth of heat penetration of the pad a=5.2 mm, the dimensionless braking time $\tau_{s}=0.33$, and the temperature scaling factor $\Theta_{0}=10,373{\;}^{\circ }C$, based on Equations (10)–(12).

**Figure 3:**
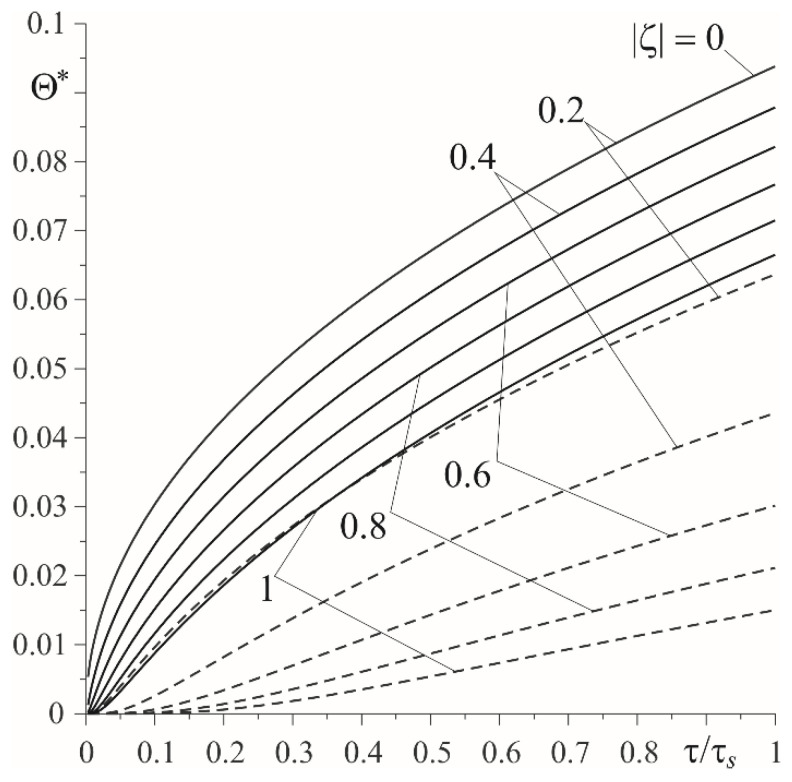
Evolutions of dimensionless temperature rise $\Theta^{*}(\zeta ,\tau )$ on the established distances |ζ| from the friction surface during sliding with a constant velocity: cast iron—solid lines; FGM—dashed lines.

**Figure 4:**
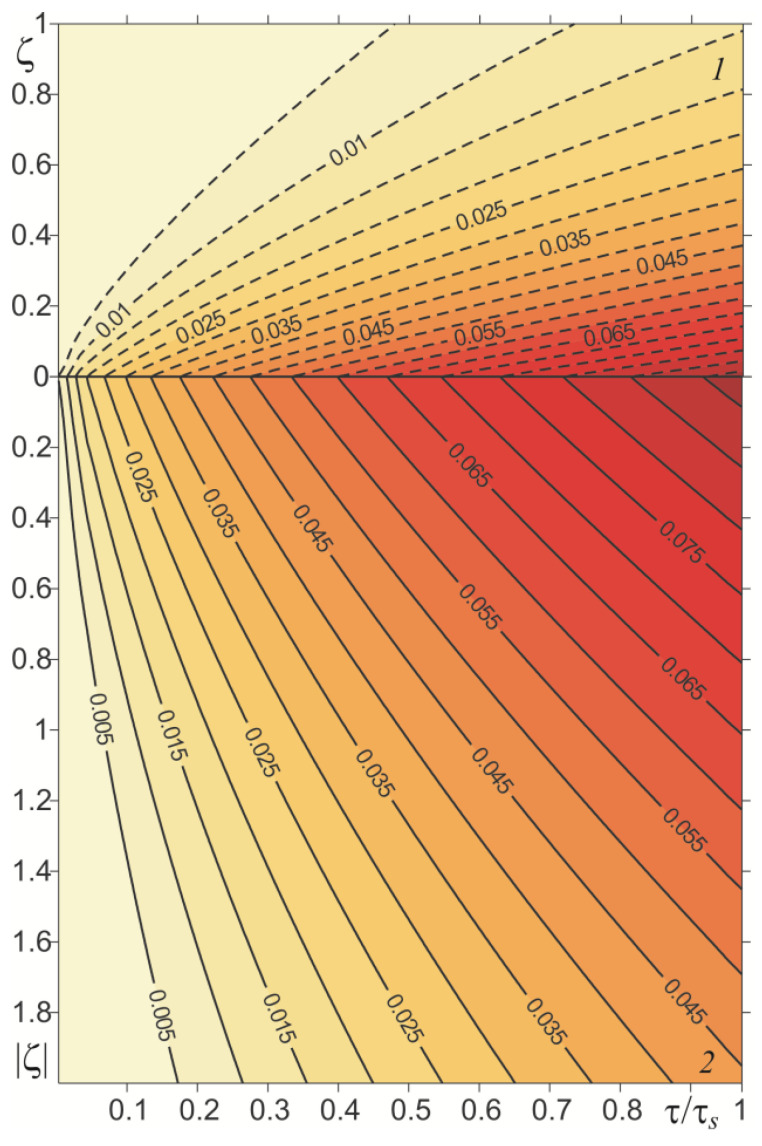
Isotherms of dimensionless temperature rise $\Theta^{*}(\zeta ,\tau )$ during sliding with constant velocity: cast iron (*1*)—solid lines; FGM (*2*)—dashed lines.

**Figure 5:**
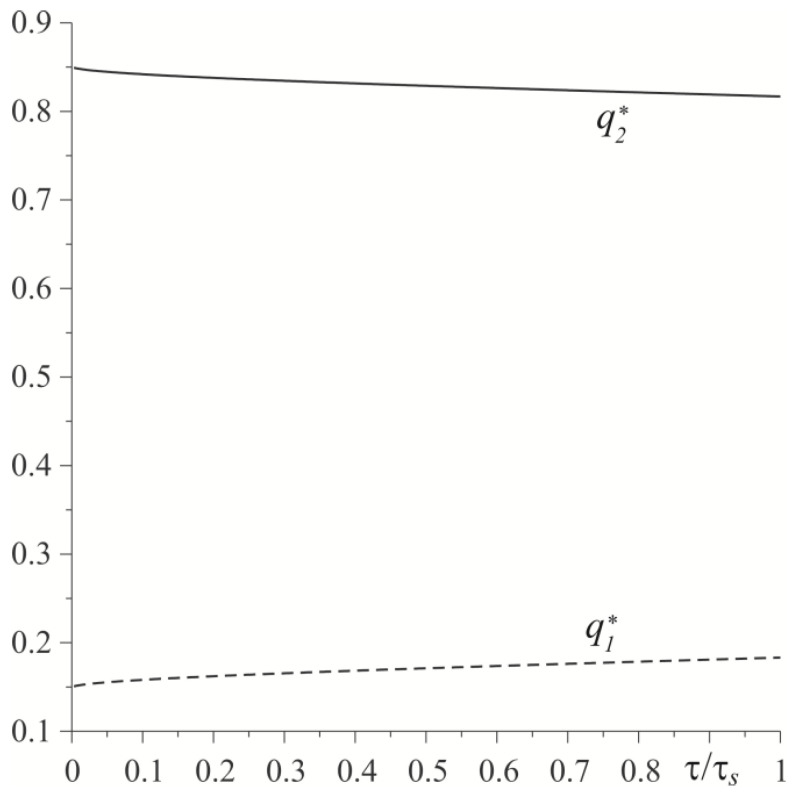
Evolutions of dimensionless intensities of heat fluxes $q_{l}^{*},\;l=1,2$, directed along the normal from the friction surface to the insides of the elements made of cast iron (solid line) and FGM (dashed line) under uniform sliding.

**Figure 6:**
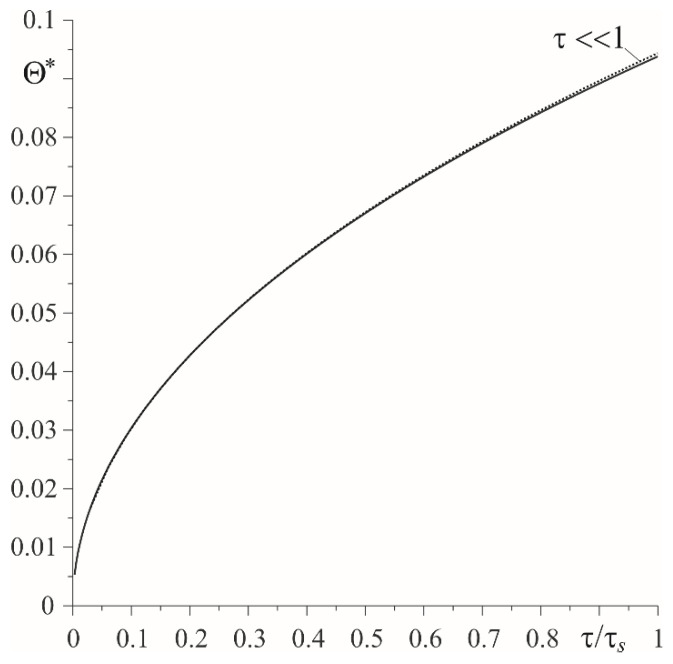
Change with time of the dimensionless temperature rise $\Theta^{*}(\tau )$ on the friction surface ζ=0 during sliding with constant velocity: exact solution—solid line; asymptotic solution—dashed line.

The integrals in the obtained solutions were calculated numerically using the QAGI procedure of the QUADPACK package [15]. Changes of the dimensionless temperature rise and intensities of heat fluxes during sliding with a constant velocity are presented in Figure 3, Figure 4, Figure 5 and Figure 6.

Temporal profiles of the dimensionless temperature rise $\Theta^{*}(\zeta ,\tau )$ (49)–(53) at a few distances from the friction surface are shown in Figure 3. The temperature of both elements increased monotonically over time. The highest temperature was reached on the friction surface, and decreasing moving away from it. For a given distance from this surface, the temperature of the homogeneous cast iron element was always higher than the temperature of the functionally graded element. Having a much greater thermal conductivity, the cast iron was heated to a much deeper extent than the FGM (Figure 4).

Temporal profiles of dimensionless heat flux intensities $q_{l}^{*}(\tau )$, l=1,2 (59), (60) are demonstrated in Figure 5. It was found that the main element that absorbs frictional heat is the cast-iron disc, especially at the initial stage of the heating process. The amount of heat directed from the friction surface towards the inside of the pad increases with time, and towards the inside of the disc it decreases. A comparison of dimensionless temperature values $\Theta^{*}(\tau )$ of the friction surface, found by means of the exact (54), (55) and asymptotic solutions (74), (75) are shown in Figure 6. In the considered range of Fourier number $0\leq \tau \leq \tau_{s}$, the respective temperature values were almost the same.

**Figure 7:**
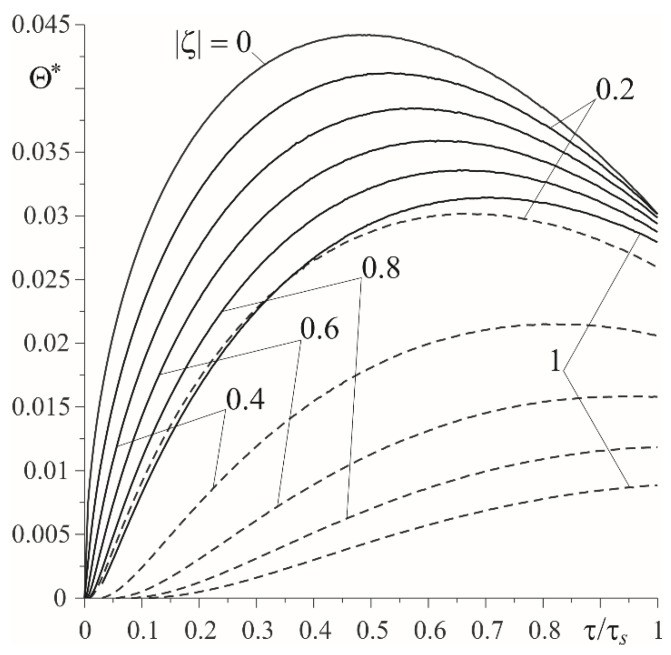
Evolutions of dimensionless temperature rise ${\hat{\Theta }}^{*}(\zeta ,\tau )$ on the established distances |ζ| from the friction surface during braking with constant deceleration: cast iron—solid lines; FGM—dashed lines.

**Figure 8:**
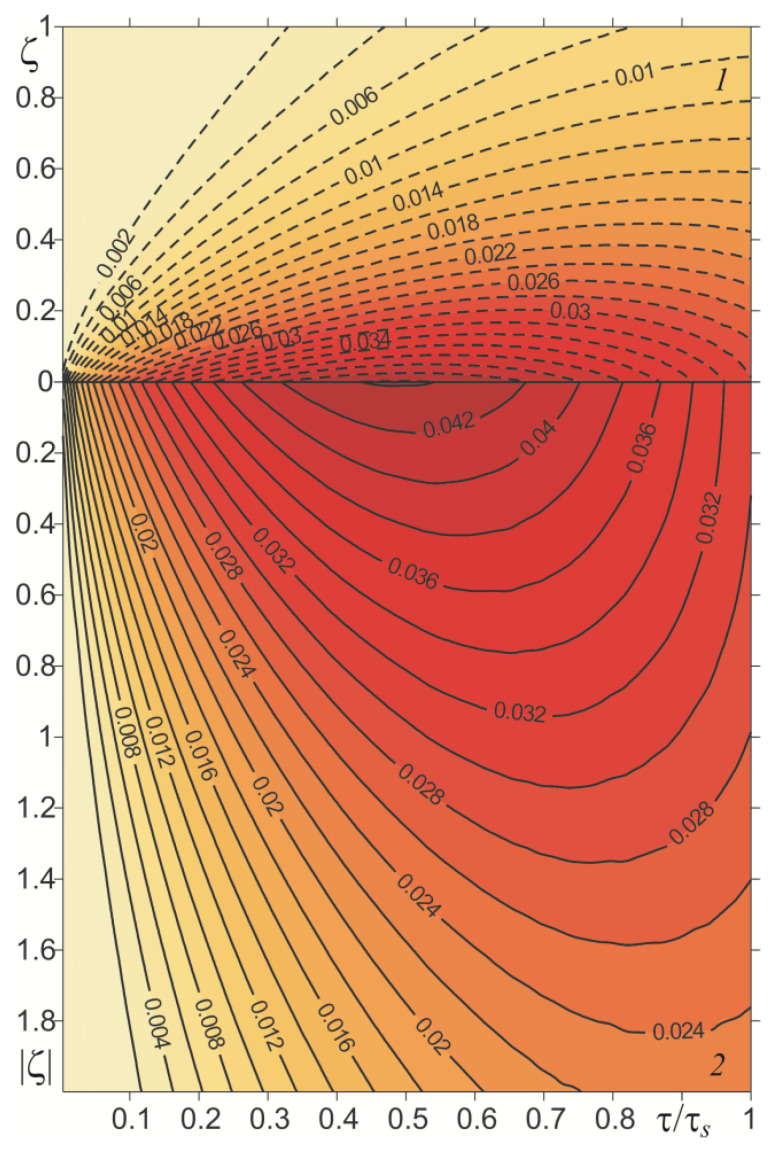
Isotherms of dimensionless temperature rise ${\hat{\Theta }}^{*}(\zeta ,\tau )$ during braking with constant deceleration: cast iron (*1*)—solid lines; FGM (*2*)—dashed lines.

**Figure 9:**
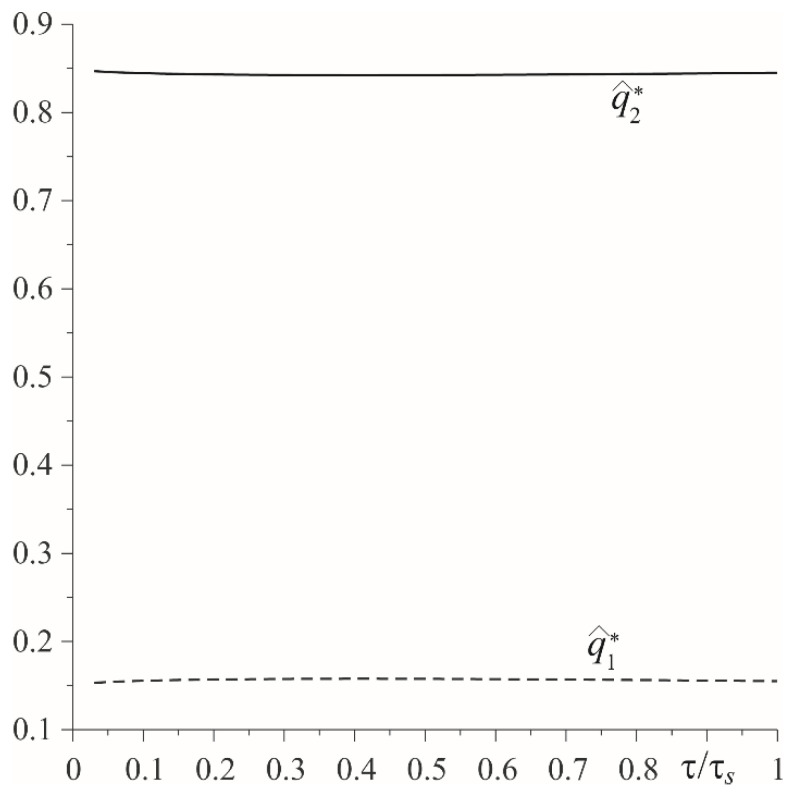
Evolutions of dimensionless intensities of heat fluxes ${\hat{q}}_{l}^{*},\;l=1,2$ directed along the normal from the friction surface to the insides of the elements made of cast iron (solid line) and FGM (dashed line) during braking with constant deceleration.

**Figure 10:**
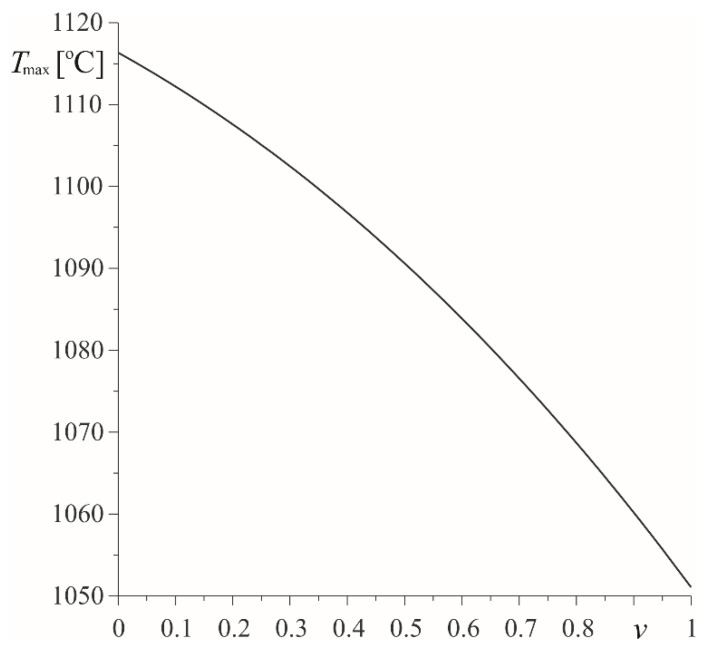
Dependency of maximum temperature $T_{\max}$ during braking with constant deceleration on the volumetric fraction v.

Relevant results, obtained in the case of a linearly decreasing velocity (so-called braking with a constant deceleration), are presented in Figure 7, Figure 8, Figure 9 and Figure 10. The temporal profile of the dimensionless temperature rise ${\hat{\Theta }}^{*}(\zeta ,\tau )$ (87)–(89) during the braking process was different than during uniform sliding (Figure 7). The dimensionless time to reach the maximum temperature on the friction surface was $\tau_{\max}\approx 0.5\tau_{s}$ and became higher when increasing the distance from it. After reaching the maximum value, the temperature dropped. More vividly, such a concentration of high temperature near the friction surface is shown in the distribution of isotherms, as illustrated in Figure 8. Apparently, as in the case of uniform sliding, the greater part of the frictionally-generated heat is absorbed by the cast iron disc (≈85%) (Figure 9). The intensities of heat fluxes ${\hat{q}}_{l}^{*}(\tau )$, l=1,2 (91) are almost unchanged during the entire braking process.

The results presented in Figure 3, Figure 4, Figure 5, Figure 6, Figure 7, Figure 8 and Figure 9 were obtained with the same (v=0.5) volumetric components fractions of ZrO_2_ and Ti-6Al-4V, determining the effective specific heat capacity and density using formula (9). On the other hand, the change of the maximum temperature $T_{\max}\equiv T(0,t_{\max})$ with the increase of the parameter v is presented in Figure 10. The highest value $T_{\max}=1117{\;}^{\circ }C$ was achieved in the case of the pad made of pure zirconium dioxide, and the lowest $T_{\max}=1052{\;}^{\circ }C$, when it was made of the titanium alloy.

## 7. Conclusions

A mathematical model was proposed to determine the transient temperature field in a friction pair, in which one element is made of a functionally graded material and the other is made of a homogeneous material. It was assumed that the thermal conductivity of a FGM increases exponentially with the distance from the contact surface. An exact solution of the appropriate boundary–value problem of heat conduction was formulated and then solved, with consideration of frictional heat generation. Two cases of the friction power temporal profiles were analyzed in detail: constant (uniform sliding), and linearly decreasing in time (braking with constant deceleration). A numerical analysis was performed for a two-component FGM (ZrO_2_ + Ti-6Al-4V) sliding on the cast-iron disc. It was found that the greater part of heat generated due to friction was absorbed by the cast iron (about 85%), which resulted in a greater depth of effective heat penetration in this element, due to the high thermal conductivity of cast iron. At a fixed distance from the friction surface, the temperature of the cast iron element is higher than that of the FGM element, in both the considered cases: uniform sliding, and during braking. Thus, in order to protect systems against such undesirable phenomena as overheating and thermal cracking etc., the use of FGM on the friction elements may be justified. It is also worth emphasizing that in the analyzed range of the Fourier number change 0≤τ≤0.33, the appropriate asymptotic solution can be effectively used, giving a high accuracy of calculations, without the inconveniences related to numerical integration in an exact solution.

It should be noted that the shape of the friction pair elements, as well as their positional relationship, can be considered in some spatial problems of friction solved by numerical methods, in particular the finite element method (FEM). The temperature evolution obtained by them oscillates, as a result of the heating area moving on the surface of the brake disc. The model proposed in this paper is one-dimensional, based on a physically-justified assumption that heat, generated by friction of two elements, propagates in the direction perpendicular to the contact surface. This allows determining the mean temperature (from the above-mentioned oscillations) on the friction surfaces of both elements.

According to the current state of knowledge [16,17], the temperature of the friction surface is the sum of the volume temperature (average temperature in volume), the mean temperature, and the flash temperature. The flash temperature is the component that takes into consideration the texture of the friction surfaces. The flash temperature calculation models need appropriate experimental data as input parameters. In the case of homogeneous materials, such data can be found in the article in ref. [13]. However, we have not found such data for the considered friction pair. The development of models for determining the flash temperature of such couples is a potential direction for our research. In the future, we intend to expand the proposed mathematical model with the possibility of testing the temperature of friction systems of this type (functionally graded and homogeneous materials) made of thermally sensitive materials and the temperature-dependent friction coefficient.

## Figures and Tables

**Table 1:** Material properties at the initial temperature $T_{0}$ [3,13].

Material	$\begin{array}{c} \mathrm{Thermal}\;\mathrm{Conductivity}\\ {\mathrm{Wm}}^{-1}K^{-1} \end{array}$	$\begin{array}{c} \mathrm{Specific}\;\mathrm{Heat}\;\mathrm{Capacity}\\ J\;{\mathrm{kg}}^{-1}K^{-1} \end{array}$	$\mathrm{Density}\;\;\mathrm{kg}\;m^{-3}$
ZrO_2_	1.94	452.83	6102.16
Ti-6Al-4V	6.87	538.08	4431.79
ChNMKh	52.17	444.6	7100

**Table 2:** Input parameters [14].

$\mathrm{Friction}\;\;\mathrm{Coefficient}\;f_{0}$	$\mathrm{Nominal}\;\mathrm{Pressure}\;p_{0}^{},\;\mathrm{MPa}$	$\mathrm{Initial}\;\mathrm{Sliding}\;\mathrm{Speed}\;V_{0}^{},\;{\mathrm{ms}}^{-1}$	$\mathrm{Initial}\;\mathrm{Kinetic}\;\mathrm{Energy}\;W_{0}^{},\;\mathrm{kJ}$	$\mathrm{Nominal}\;\mathrm{Contact}\;\mathrm{Area}\;A_{a},\;m^{2}$	$\mathrm{Initial}\;\;\mathrm{Temperature}T_{0}^{},{}^{\circ}C$
0.27	0.602	23.8	103.54	0.00221	20

## Data Availability

No new data were created or analyzed in this study. Data sharing is not applicable to this article.

## Nomenclature

a	Effective depth of heat penetration (m)
$A_{a}$	Area of the nominal contact region (m^2^)
c	Specific heat capacity (J kg^−1^ K^−1^)
$f_{0}$	Coefficient of friction (dimensionless)
$I_{k}(\cdot )$	Modified Bessel functions of the first kind of the *k*th order
$J_{k}(\cdot )$	Bessel functions of the first kind of the *k*th order
k	Thermal diffusivity (m^2^ s^−1^)
K	Thermal conductivity (W m^−1^ K^−1^)
p	Dimensionless parameter of the Laplace integral transform
$p_{0}$	Nominal value of the contact pressure (Pa)
r	Radial coordinate in the polar system (m)
R	Radius of integration contour (m)
q	Specific power of friction (W m^−2^)
$q_{0}$	Nominal value of the specific power of friction (W m^−2^)
t	Time (s)
$t_{s}$	Stop time (s)
T	Temperature (${}^{\circ }C$)
$T_{0}$	Initial temperature (${}^{\circ }C$)
v	Volume fraction of the material phases (dimensionless)
$V_{0}$	Initial velocity (m s^−1^)
$W_{0}$	Initial kinetic energy of the system (J)
z	Spatial coordinate in axial direction (m)
**Greek Symbols**	
γ	Parameter of material gradient (m^−1^)
$\gamma^{*}$	Dimensionless parameter of material gradient
Γ	Integration contour
δ	Radius of integration contour (m)
Θ	Temperature rise (${}^{\circ }C$)
$\Theta^{*}$	Dimensionless temperature rise
$\Theta_{0}$	Temperature rise scaling factor (${}^{\circ }C$)
${\bar{\Theta }}^{*}$	Dimensionless transform of temperature rise
ρ	Density (kg m^−3^)
τ	Dimensionless time
$\tau_{s}$	Dimensionless time of braking
ζ	Dimensionless spatial coordinate in axial direction
φ	Angular coordinate in the polar system (rad)
